# The Extraordinary Nature of Barney's Drumming: A Complementary Study of Ordinary Noise Making in Chimpanzees

**DOI:** 10.3389/fnins.2017.00002

**Published:** 2017-01-19

**Authors:** Valérie Dufour, Cristian Pasquaretta, Pierre Gayet, Elisabeth H. M. Sterck

**Affiliations:** ^1^Ethology Evolutive Team, Institute Pluridisciplinaire Hubert Curien (IPHC), University of Strasbourg, CNRSStrasbourg, France; ^2^Research Center on Animal Cognition (CRCA), Center for Integrative Biology (CBI), Centre National de la Recherche Scientifique, University of Toulouse, UPSToulouse, France; ^3^Ethology Research, Animal Science Department, Biomedical Primate Research CenterRijswijk, Netherlands; ^4^Animal Ecology, Utrecht UniversityUtrecht, Netherlands

**Keywords:** music, drumming, object manipulation, chimpanzees, Barney's colony

## Abstract

In a previous study (Dufour et al., 2015) we reported the unusual characteristics of the drumming performance of a chimpanzee named Barney. His sound production, several sequences of repeated drumming on an up-turned plastic barrel, shared features typical for human musical drumming: it was rhythmical, decontextualized, and well controlled by the chimpanzee. This type of performance raises questions about the origins of our musicality. Here we recorded spontaneously occurring events of sound production with objects in Barney's colony. First we collected data on the duration of sound making. Here we examined whether (i) the context in which objects were used for sound production, (ii) the sex of the producer, (iii) the medium, and (iv) the technique used for sound production had any effect on the duration of sound making. Interestingly, duration of drumming differed across contexts, sex, and techniques. Then we filmed as many events as possible to increase our chances of recording sequences that would be musically similar to Barney's performance in the original study. We filmed several long productions that were rhythmically interesting. However, none fully met the criteria of musical sound production, as previously reported for Barney.

## Introduction

The universality of music across human cultures is an undisputable fact: all humans make music, sing, dance, and gather to enjoy sharing emotions elicited by musical performances (Merker et al., 2015). By contrast, there is very limited evidence that our closest living relatives, the great apes, make music in the same way (Fitch, 2006). From an evolutionary perspective, this leaves us wondering about the origins of our musical skills. Archeological remains do not provide sufficient evidence of instruments being used to create music prior to 40,000 BC, nor do they suggest the presence of any other musical behaviors (Kunej and Turk, 2000). Great apes probably possess some prerequisites for musical productions (Fitch, 2006; Honing et al., 2015). Drumming in chimpanzees and chest beating in gorillas is considered a homolog to human music: a shared ancestral trait not found in less closely related species (Fitch, 2006). Thus, taking a closer look at great ape drumming behavior may enlighten our understanding of our own musicality.

Chimpanzees drum on tree buttresses or other resonant structures as part of their dominance displays (Goodall, 1986). The chimpanzee Mike was observed repeatedly charging higher-ranking males whilst propelling kerosene cans in front of him (Goodall, 1986). Drumming can accompany vocal signals such as long-distance calls (e.g., climax of a pant hoot), sometimes even replacing part of the vocal phrase (Boesch, 1991; Arcadi, 1996; Babiszewska et al., 2015). This combination of vocalizations and noise making using objects is generally associated with social tension or high levels of arousal within the group (Goodall, 1986; Nishida et al., 1999). Other individuals can join the initiator by vocalizing, drumming, or both (Fedurek et al., 2013). Also gorillas drum on their own bodies and objects during displays (Schaller, 1963). Generally, drumming in display contexts is likely to be perceived by others as a demonstration of strength.

Drumming, leaf clipping, and breaking or shaking branches are examples of how chimpanzee males create sound with objects to communicate sexual interest (Nishida, 1980; Goodall, 1986). After demonstrating such intent, it is not rare to see the targeted female approach and present for a mount. Similarly, musically qualified humans may be more successful at seduction than their non-musical counterparts, indicating that musical ability could be a sign of male health and fertility (Miller, 2000; Sluming and Manning, 2000; Charlton, 2014).

Finally, making noise can be associated with pleasurable emotion, both in humans and great apes. Humans *play* music. Young chimpanzees often drag branches noisily along the ground and were seen, on one occasion, to repeatedly hit a clay pot (Matsusaka, 2012), seemingly enjoying the noise. Thus, on both contextual and emotional levels, there are several links between sound making with objects in great apes and instrumental music in humans.

However, many other properties of noise making with objects by great apes do not meet criteria for music. Indeed, drumming in great apes is generally contextualized (sex, play, or display context), whereas humans produce music outside any particular context or function (Arom, 2000). Music is very often rhythmical, while drumming sequences of apes are generally too short to allow for rhythm to be detected (but see Dufour et al., 2015).

Another key component of music production is the performers' capacity to synchronize their actions to an external beat (Arom, 2000). We know that pinnipeds (Cook et al., 2013), cockatoos (Patel et al., 2009), parrots (Schachner et al., 2009), and budgerigars (Hasegawa et al., 2011) can learn or be trained—with varying levels of precision—to synchronize their body movement to a rhythm. A form of action entrainment by motor mimicry of pounding gestures has been reported in young chimpanzees watching others cracking nuts (Fuhrmann et al., 2014). The female chimpanzee Aï spontaneously pressed two keys on a keyboard in synchrony to a rhythmical auditory stimulus without any previous training (Hattori et al., 2013). Recently, a bonobo was found to occasionally match its own drumming tempo to the one of a human drummer (Large and Gray, 2015), even when the tempo differed slightly from the natural pace of the bonobo. However, the tempo matching disappeared quickly despite the bonobo being encouraged and rewarded for drumming. By comparison, human children can synchronize to external drumming at the age of around 3 years, increasing in accuracy as they grow older (Honing et al., 2012). Kanzi, a language-trained bonobo, was reported to perform rhythmical drumming (Kugler and Savage-Rumbaugh, 2002), but there are no published data describing this event. In a recent study, we described a long drumming solo on an upturned plastic barrel by a chimpanzee named Barney. This solo was rhythmic, decontextualized, and fitted several criteria for human music (Dufour et al., 2015). It would be justifiable to question the significance of this unique observation: was Barney's performance a “once in a lifetime” event, i.e., a chimpanzee accidentally “discovering music”? Or was it a rare behavior that had gone unnoticed by chimpanzee specialists and had not been given the consideration it deserved, remaining unpublished due to its anecdotal nature? Most importantly, can Barney do it again?

To identify the factors leading to Barney's performance, we conducted a 2 months-long survey on sound making with objects in the chimpanzee facility where Barney was living. Our first aim was to gather information on factors influencing the duration of sound making using objects found in the environment. To that end we recorded the context, the medium used, and the complexity of sound production techniques. Furthermore, we noted the sex of the individual producing sound with objects. Reports on chimpanzee drumming in the wild (Nishida, 1980; Goodall, 1986; Arcadi, 1996; Nishida et al., 1999) suggest that—in particular male chimpanzees—drum primarily in socio- or emotionally negative contexts, such as displays. However, since studies on chimpanzee drumming are rather scarce so far, formulating informed predictions for the effects of context, sex, technique, and medium on drumming durations is not straightforward. Therefore, we adapted an explorative approach to tackle potential effects of these predictor variables on the duration of sound production. Secondly, we aimed at recording as many sound production events as possible in order to detect any performances resembling Barney's original drumming (i.e., long, rhythmically interesting, and decontextualized sequences). Any such cases were checked for evidence for musicality.

## Methods

### Subjects and study site

**Figure 1:**
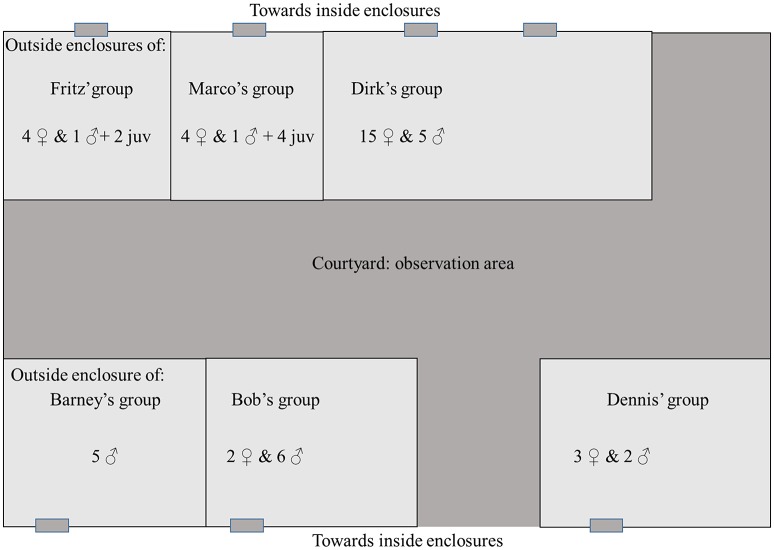
**Disposition and group composition of the chimpanzee's colony**. Some group can see each other, and all groups can hear each other.

This study took place at the Biomedical Primate Research Centre (BPRC) at Rijswijk, the Netherlands, in July and August 2005. The facility held a total of 54 individuals of which 28 were adult females and 26 adult males. The population was composed of six groups living in enclosures with outside areas facing the same courtyard (see Figure 1). Thus, some groups (those who faced other groups) could see each other and all groups could hear each other. Note that this colony was moved to the Safari Park Beekse Bergen in 2006, and that the BPRC no longer houses chimpanzees. The survey of sound production using objects or other enclosure elements as a resonating medium (sound-object use, hereafter referred to as So-U) was carried out in two phases. In Phase 1 (from 13th July to 12th August 2005) data were collected for the four groups that had at least two adult males (Dirk's group, Barney's group, Bob's group and Dennis' group). Due to low rates of So-U in one of the groups (Bob's group), this group was removed from Phase 1 data collection after a few days. In Phase 2 data collection on So-U sequence recordings took place on all six groups from the 22nd of July to the 25th of August 2005.

### Phase 1: contexts of So-U

#### Data collection

In Phase 1 the goal was to gather as much information about the factors affecting the duration of So-U as possible. One observer (PG) stood in the central courtyard and focused on one group for 15 min at a time. We conducted a total of 162 focal observations (54 per group). Focal observations were spread equally throughout the day (from 8.30 to 10 am: 15 observations per group, 10.30–12.20: 20 observations per group, and 1.20–5 p.m.: 19 observations per group) and the groups were observed in random order within each time period. During focal group observations the observer wrote down all occurrences of So-U in the group, noting the identity of the individual producing the sound, the duration of So-U, the type of medium used, the technique used, and any information (individual posture, behavior, or external factors) that could indicate the context of occurrence (Table 1). Some contexts were easily identifiable (i.e., nest-building, play, sexual activity, intimidation display without aggression, aggression, see Goodall, 1986). Others were not always clearly discernable in the absence of any obvious contextual information. An example for this is “tension,” which was defined as So-U when the animal had its hair erect (hunched back posture) without escalation into a display or an aggression and in the absence of any other contextual element. Indeed, hunching can sometimes also occur in courtship (but lead then to copulation or to attempted copulation, accompanied by an erected penis), greetings (involving a “friendly reunion”), or excitement (upon seeing enriching food, hearing other chimpanzees, etc.) (Nishida et al., 1999). When courtship and greeting could be excluded and no external stimuli potentially triggering excitement were apparent, we assigned the context “tension” to the So-U episode.

**Table 1:** **Description of the behavioral units recorded live by the observer for contexts, type of medium, techniques, vocalizations, and postures**.

**CONTEXTS**	
Tension	An individual produces a So-U, with hunched back while standing or sitting, but with no agitated movements, display, or aggression. Often with soft hoots or rising hoots.
Display	An individual produces a So-U, with hunched back accompanied by bipedal stamp, and/or agitated movements but no aggression. Display can involve an initial "tension" that becomes a display, with rising hoot and/or climax vocalizations.
Aggression	An individual produces a So-U that is immediately followed or preceded by a chase of another individual, or the sound-maker is himself attacked or chased by a member of his group. Aggression can involve initial tension or display but they escalate into aggression, often with vocalizations.
Nest-building	So-U performed while an individual is regrouping together several substrates that are manipulated repeatedly around the body. The individual is otherwise immobile, generally sitting, with no hunched back and no play face.
Sexual activity	So-U performed by an individual who is looking at a female with penis erected, generally followed by the approach of a female who presents her genitalia to the male; sometimes followed by a mount.
Attention-seeking	So-U performed by an individual who is trying to attract the attention of keepers, the observer, or neighboring chimpanzees by knocking on medium or and clapping, then checking for a reaction; can be repeated if no reaction is obtained.
Play	So-U performed by an individual who is displaying a play-face but no hunched back, often accompanied by energetic movements.
Teasing	So-U performed in a context where the sound-maker uses a medium to approach and tease another individual. No chase or hunchback from the teaser is involved, a play face can be emitted by the teaser but not by the target.
Outside group noise	So-U produced after the occurrence of unusual noise or unusual colony activity (for example, several chimpanzees from outside the group vocalizing or displaying).
None	None of the above contexts.
**TYPE OF MEDIUM**	
Furniture, small object	Hanging tires, bench, flaps, and concrete wall, plastic bottles, paper, cardboard
Plastic container	Small container, half or large barrel.
Metallic part of the enclosure	Fence, doors, metallic ground.
**TECHNIQUE ELABORATION**	
Simple technique	
Hit or hold & hit	Hits a medium once or repeatedly (either soft or strong hit), using feet or hands.
Hold & trail	Holds a medium with one or both hands and repeatedly trails it on a surface (ground or enclosure walls).
Hold & shake	Holds a medium with one or both hands and repeatedly shakes it, can also occur with substrate placed on against a surface (ground or enclosure walls).
Hold & push	Holds a medium and pushes it over a distance while walking or running.
Hold & ½ circle	Holds a medium with one hand and produces a large semi-circular movement from one side to the other, making the object hit the wall on either side.
Complex technique	A combination of several of the above techniques.
Vocalizations	Soft hoot, rising hoot, climax vocalization, other.
Postures	Sitting, standing, hunched back, bipedal stamp, bipedal run, bipedal walk, rocking.

#### Data re-coding for phase 1

To ensure a sufficiently large and reliable dataset allowing for So-U analysis we introduced categories for contexts, drumming techniques, and objects used for sound production respectively.

**Figure 2:**
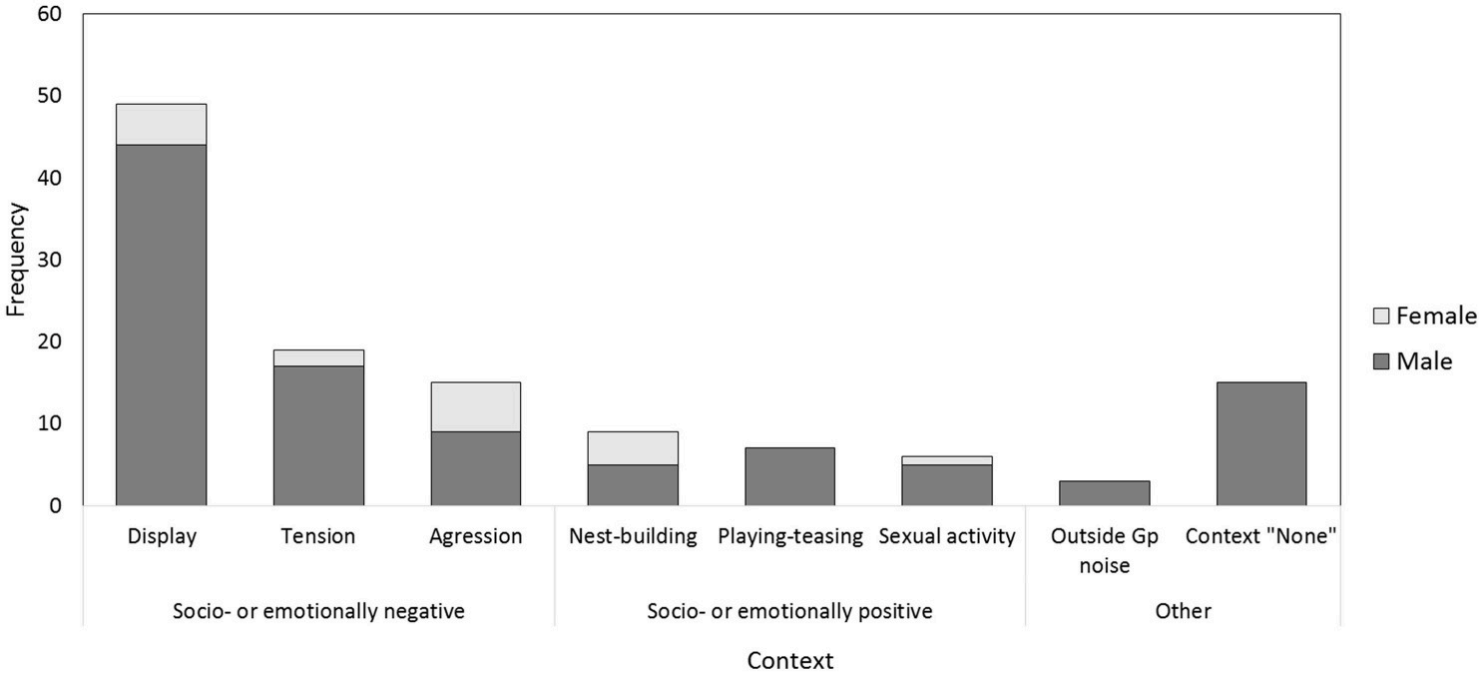
**Contexts of So-U according to sex**. Main contexts are aggression, display, tension (socio- or emotionally negative contexts). Other contexts are sexual activity, teasing, playing, attention seeking, and nest-building (considered as socio- or emotionally positive). Note that most So-U in sexual activity are initiated by males, except one initiated by a female who sought the male attention by knocking on a door. So-U with unidentified contexts (context “none”) are also recorded.

As So-U occurred frequently when individuals were tense, aggressive, or displayed we regrouped these contexts into one category that we termed “socio- or emotionally negative contexts.” Socio- or emotionally positive contexts comprised sexual activity, playing, teasing, nest-building, and attention seeking. Any outside-group noise and unidentified contexts (Figure 2) were excluded from data analyses. Note that comparing So-U durations between contexts when working with data that were not controlled for durations of the behavioral category itself might be questionable. We took this limitation into account when discussing the results.

For the noise production techniques we distinguished between “simple” (only one technique used, i.e., hitting, shaking, trailing, half-circling, pushing, or throwing) and “complex” (combination of two or more techniques) techniques.

Regarding the objects used to produce noise we differentiated between (i) metallic media (doors, fence, metallic ground), (ii) plastic containers (small plastic containers, half and large plastic barrels), and (iii) small or non-resonating objects (small plastic bottles, enclosure furniture, cardboard tubes).

#### Statistical models for phase 1

First we used log likelihood ratio test (LRT) to check for individual differences in the frequency and handling duration of So-U. We compared the full model with individual nested in group as a random effect against the full model with only group as a random effect. We build a model to study the response variable “duration of So-U.” We used multi-model inference and model averaging methods to calculate the weight of evidence (Wi) for each predictor involved in the model (Burnham and Anderson, 2002). We standardized the binary predictors to a common scale both to correctly estimate their influence (Engqvist, 2005) and to control for some possible degree of collinearity among them Schielzeth (2010). We carried out an information theoretical approach: all the possible models were run and ranked based on their AIC scores (Akaike, 1985) after correction for small sample sizes (AICc) and their normalized Akaike weights (AICw, Burnham and Anderson, 2002). We computed estimates, standard errors and 95% confidence intervals (CI) for models whose cumulative model weights reached 90% of the total weights. We ran model selections and averages using the R Package MuMIn (Barton, 2009). The model was run following a negative binomial error distribution using the R package glmmADMB (Fournier et al., 2012).

The full model included the following predictor variables: (i) the medium, (ii) the complexity of the technique, (iii) the context, (iv) the sex, and (v) all possible interactions of these main effects except for the interaction “technique-medium.” Indeed, among all the So-U performed with mixed techniques (33 events) 32 occurred with the same medium (plastic container) preventing us to investigate the effect of this interaction. Note that only one female used a small medium (and in only one occasion). Therefore, we also excluded the interaction “sex-medium” from the model.

### Phase 2: video recording of So-U for further rhythmical analysis

In Phase 2 we conducted all occurrence sampling of So-U in all six groups to increase our chances of filming a long, decontextualized, and rhythmically complex So-U resembling Barney's original performance. We recorded the identity of the performer and the context of occurrence. For interesting bouts rhythmical analysis was conducted in the same way as in Dufour et al. (2015), i.e., by checking the possibility of rhythmical patterns in a series of impacts (Ljung-box test analysis), and then checking the predictability of the next section using autocorrelation analysis (see Supplementary Methods). For each sequence data were analyzed using R (R Core Team, 2013). *P* level of significance was set at 0.05.

## Ethics statement

The study was conducted in compliance with all relevant Dutch laws and respected international and scientific standards and guidelines. All analyses were based on the recording of spontaneous behavioral sequences initiated by the chimpanzees. Due to the observational nature of this study and the absence of discomfort for the animals no additional permission was required from the institute's animal experiment committee, as assessed by the Biomedical Primate Research Centre Animal welfare officer.

## Results

### Phase 1: contexts of So-U

During focal observations in Phase 1 we monitored a total of 123 So-U with various media (Supplementary Figure 1). So-U occurred in several contexts including aggression, display, tension, sexual activity, teasing, playing, attention seeking, nest-building, and outside group noise. 15 (of the 123) bouts could not be assigned to any context. Sound production in unidentified contexts occurred only in males (Figure 2). While So-U occurred both in males and in females, males did so more often than females (males: 105 times, females: 18 times), all contexts included. We recorded 88 So-U with simple techniques and 35 with complex techniques (16 individuals out of 24 never used complex techniques). Focusing on the two main contextual categories (thus excluding context “none” and context “outside group noise”), we recorded more So-U in socio- or emotionally negative contexts (83) than in socio- or emotionally positive contexts (22). There were significant differences between individuals both in the frequency and duration of So-U (LRT frequency model: *df* = 1, Δ deviance = 4.25, *P* = 0.039; LRT duration model: *df* = 1, Δ Deviance = 6.254, *P* = 0.012).

**Figure 3:**
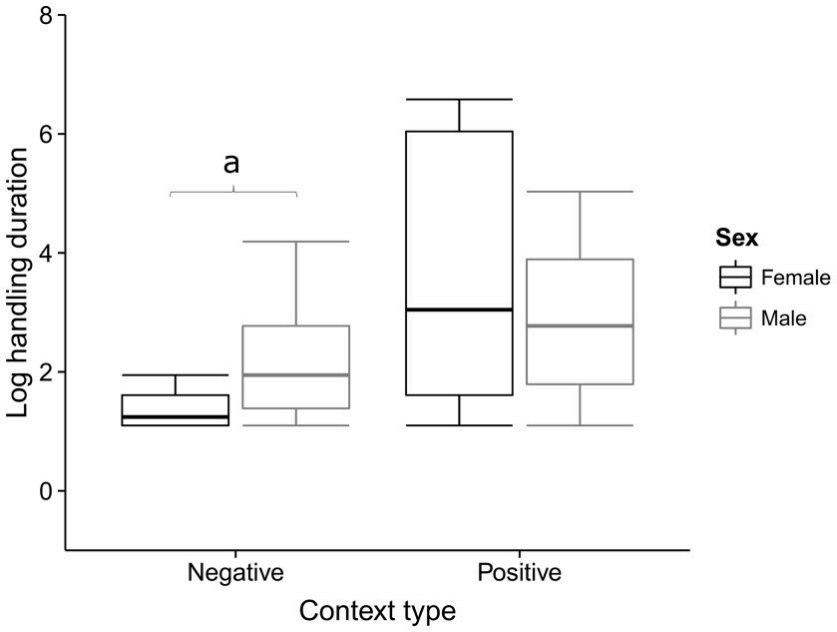
**So-U duration according to the context for males and females**. The average model performed on the duration of So-U indicates that males had significantly longer So-U than females in the negative contexts (“a” indicates this significant influence in the figure).

**Figure 4:**
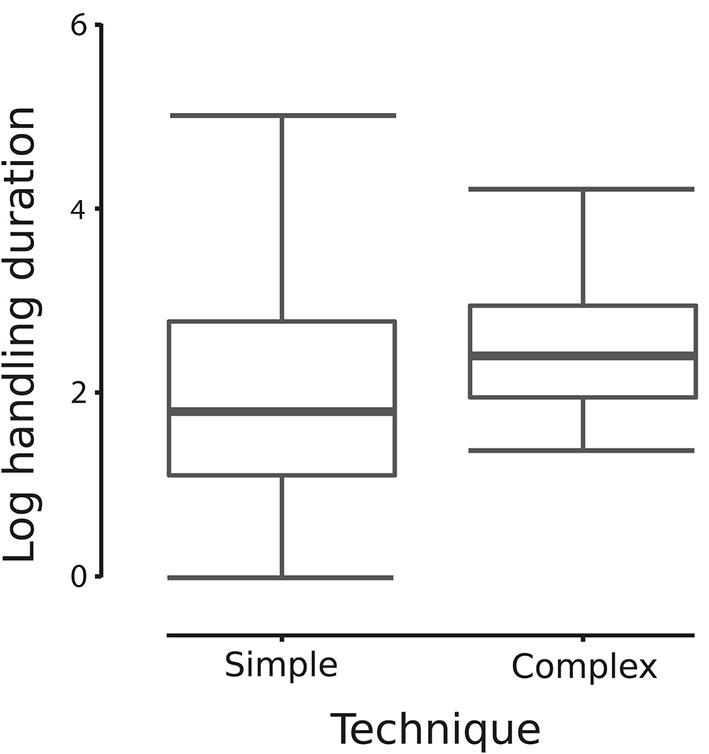
**So-U duration according to each type of technique (complex or simple)**. The average model indicates that complex techniques involved longer durations than simple techniques (“a” indicates this significant influence in the figure).

The time spent handling a medium in So-U varied from 1 to 720 s, with a median duration of 6 s. Model averaging on the duration of So-U as the response variable revealed that context, sex, the interaction between context and sex, and the technique used had higher relative importance than other variables in the model (Table 2). So-U in socio- or emotionally positive contexts lasted longer than in socio- or emotionally negative ones (Estimate = 1.053, 95% IC: 0.052–2.055; Table 2). Males had longer So-U than females but this was only significant in negative contexts (in negative contexts: Estimate = −3.253, 95% IC: −4.891 to −1.615; in positive contexts: Estimate = −1.991, 95% IC: −4.151–0.167; Table 2, Figure 3). Complex techniques lasted longer than simple ones (Estimate = 1.053, 95% IC: 0.132–1.161; Figure 4).

**Table 2:** **Model average using AICc-based selection approach, showing estimate, standard error (SE), 95% confidence interval (95% CI) and relative weight of evidence (Wi) for each variable both for the handling duration model**.

	**Estimate**	**SE**	**95% CI**	**Wi**
(Intercept)	1.948	0.357	1.242 to 2.653	
Medium Psml	0.647	0.289	0.074 to 1.219	0.82
Medium Pt	0.665	0.359	−0.046 to 1.377	
Technique complex	0.647	0.26	**0.132 to 1.161**	**0.95**
Sex male	0.652	0.452	−0.245 to 1.549	1
Context positive	1.053	0.505	**0.052 to 2.055**	**1**
Technique complex: context positive	−0.97	0.593	−2.147 to 0.207	0.46
Sex male: context positive	−3.259	0.823	−**4.892 to −1.626**	**1**
Medium Psml: context positive	−0.21	0.807	−1.811 to 1.391	0.37
Medium Pt: context positive	1.148	0.793	−0.426 to 2.723	
Technique complex: sex male	0.235	0.737	−1.229 to 1.698	0.15

*The model was run using a “poisson” distribution and included (i) the medium, (ii) the complexity of the technique, (iii) the context, (iv) the sex, and (v) all possible interactions of these main effects (except for the interaction “technique-medium” and “sex-medium,” see methods) as fixed effects and individual nested in group as random effect. Estimates with 95% (CI) that don't overlap 0 indicates a significant influence on the response variable (highlighted in bold)*.

### Are there any similarities with Barney's drumming?

In Phase 1, we observed 27 So-U that lasted longer than 20 s. Eight were performed with either a half barrel or a large barrel. Only one of these performances was not clearly associated to a context, as the performer alternated between hoots and a relaxed face while shaking the barrel. The seven remaining bouts of sound production were contextualized (4 displays, 1 tension, 1 nesting, 1 sexual activity). Barney performed only six So-U, two of which involved using a small container, and four of which involved hitting a door. Five of these events occurred in a tension context and one was associated to a display.

In Phase 2, we filmed 262 events of So-U by various individuals, including 90 So-U that lasted longer than 20 s. Eighty-four of these occurred in a clearly recognizable context: socially negative (46), nest-building (10), sexual activity (9), attention-seeking (3), or play (16). Note that several sequences could sometimes be detected within one So-U bout. In total we analyzed the rhythmical properties of 10 So-U, producing a total of 20 sequences, i.e., those in which successive beats could be clearly identified from the background noise (Table 3). Rhythmical analysis concerned only seven contextualized So-U (Table 3). Two of these were socially negative So-U (Oscar seq 1; Oscar seq 2.1 and 2.2, Table 3). However, the technique used in these cases (holding a barrel and hitting it against the wall with half-circling trajectories, Supplementary Video 1), could potentially explain their rhythmicity. Three sexual displays (Paul seq 1.1, 1.2, and 1.3: Supplementary Video 2, Dennis seq 1 and Dennis seq 2.1 and 2.2) showed interesting and complex rhythmical patterns (Ljung-box portmanteau test, Supplementary Figure 2). In Paul seq 1.2, for example, there were alternating series of short and long inter-beat durations with a remarkably long-term dependency (autocorrelation test, Table 3 and Supplementary Figure 3). The tempo was independent of the technique or medium used, and seemed to be controlled by the chimpanzees. Finally, among the six So-U without clear contexts, two showed analyzable rhythmical properties (Table 3). All of them could also be rhythmically highly dependent on the technique used (see Supplementary Videos 3 and 4).

**Table 3:** **List of the 10 So-U in which a total of 20 sequences longer than 20 inter-beat intervals could be detected**.

		**LJUNG-BOX TEST**											
**So-U nbr**	**Ind & seq in So-U**	**Value**	**Khi**	***P*-value (at 1rst lag)**	**Auto-correl°**	**Mean Inter-beat (Ib) Dur° (in ms)**	**Min Ib Dur° (ms)**	**Max Ib Dur° (ms)**	**Total N° of beats**	**Context**	**Object**	**Technique**	Video^*^
1	Lenny seq 1.1	na	2.51	0.11	na	320	44	945	80	Attention-seeking	small barrel	Hold and semi-circle against the wall	AvR
1	Lenny seq 1.2	na	0.18	0.67	na	467	163	1669	21	Attention-seeking	small barrel	Hold and semi-circle against the wall	AvR
2	**Freek seq 1**	>14	8.07	**0.00**	2	284	103	1131	119	none	lock	shake	sup. vid 3
3	**Oscar seq 1**	>14	4.44	**0.04**	1	1048	606	1428	69	socially negative	half-sized barrel	Hold and semi-circle against the wall	AvR
4	**Oscar seq 2.1**	>14	10.1	**0.00**	23	1064	847	1321	200	socially negative	half-sized barrel	Hold and semi-circle against the wall	supp. vid 1 (starts at 1")
4	Oscar seq 2.2	na	0.12	0.73	na	1000	866	1263	45	socially negative	half-sized barrel	Hold and semi-circle against the wall	supp. vid 1 (3'37”)
5	Dennis seq 1	na	0.37	0.54	na	285	204	844	57	sexual	ceiling	Hit with hand	AvR
6	Paul Seq 1.1	na	0.03	0.86	na	232	171	723	22	sexual	half-sized barrel	Hit with hand	sup vid 2 (35″)
6	**Paul Seq 1.2**	>14	26.1	**0.00**	>15	242	164	382	40	sexual	big barrel	Hit with hand	sup. vid 2 (1′54″)
6	Paul Seq 1.3	na	2.05	0.15	na	312	125	837	51	sexual	half-sized barrel	Hit with hand	sup. vid 2 (2′24″)
7	Dennis Seq 2.1	na	1.03	0.31	na	519	154	1519	73	sexual	big barrel	Hit with hands	AvR
7	**Dennis Seq 2.2**	>14	11.3	**0.00**	5	517	218	1814	213	sexual	big barrel	Hit with hands	AvR
8	**Ruben Seq 1.1**	>14	8.24	**0.00**	6	414	68	1916	49	play	small barrel	Hit with hands	AvR
8	Ruben Seq 1.2	na	0.71	0.40	na	325	99	448	23	play	small barrel	Hit with hands	AvR
9	Martje seq 1.1	na	3.44	0.06	na	1072	106	1821	21	Attention-seeking	wall	Hit with feet	AvR
9	Martje seq 1.2	na	3.44	0.06	na	569	72	1775	24	Attention-seeking	wall	Hit with feet and hand	AvR
10	**Dennis seq 3.1**	>14	9.36	**0.00**	2	798	587	1223	23	none	small barrel	Hold and semi-circle against the wall	supp. vid 4 (7″)
10	**Dennis seq 3.2**	11	1.83	**0.01**	2	620	452	789	41	none	small barrel	Hold and semi-circle against the wall	sup. vid 4 (27″)
10	**Dennis seq 3.3**	>14	4.53	**0.03**	1	671	568	872	20	none	small barrel	Hold and semi-circle against the wall	sup. vid 4 (54″)
10	Dennis seq 3.4	na	0.09	0.77	na	625	491	1995	72	none	small barrel	Hold and semi-circle against the wall	sup. vid 4 (1′28″)

* *In this column the abbreviation AvR mean that associated videos or audio files are available on request to the corresponding author. Bold indicates statistically significant P-value at first lag for the ljung box test*.

## Discussion and conclusion

The evolution of musicality is shrouded in the mists of our past, but the production of sounds with objects by chimpanzees may reveal the presence of some of its prerequisites in our common ancestor. Although we did not record a second instance of decontextualized and rhythmic drumming like the one event recorded for Barney (Dufour et al., 2015), the chimpanzees of the studied colony frequently incorporated objects when making sound. Sound production occurred in a diversity of contexts. So-U produced in socially or emotionally positive contexts lasted longer than in negative contexts. The primary explanation could be that our target behaviors for socio- or emotionally negative contexts (e.g., displays) may simply not last as long as the target behaviors in socio- or emotionally positive contexts (e.g., play bouts or building a nest)—with or without So-U. Therefore, the difference in duration of So-U across contexts may likely be an artifact of socially or emotionally positive behaviors lasting longer than socio- and emotionally-negative ones. An alternative hypothesis (that may be considered in further studies) is that So-U might have lasted longer in this context because individuals were involved in a relaxing activity like nesting and playing. Their attention was maybe better focused on the production of sounds and its pleasurable aspects.

More than 85% of So-U were produced by males, which is in line with what we expected based on the literature (Nishida, 1980; Goodall, 1986; Arcadi, 1996; Nishida et al., 1999). As So-U in socio- or emotionally negative contexts lasted longer when produced by males than by females, we could speculate that male chimpanzees are more motivated than females to produce sound with object in this context, but we cannot generalized at the population level. This observation fits well with the intimidating function of buttress drumming described in wild male chimpanzees (Goodall, 1986). Further work should aim at a more detailed assessment of the communicative function of So-U by assessing responses from the audience in these contexts. In socially or emotionally positive contexts, there was no difference in object handling durations between females and males.

Finally, So-U could involve complex techniques that lasted longer than So-U with simple techniques. We hypothesize that shifting from one technique to another could be a way to counter tiredness arising from multiple repetitions of the same gestures. This illustrates how chimpanzees actively engaged in producing sounds with objects, a prerequisite for the evolution of music.

Given the diversity of contexts recorded, we cannot conclude about the main driving force in the production of So-U in our colony (388 events in <2 months). We cannot therefore pinpoint which factor most probably led to the discovery and spreading of music by our ancestors: the need to demonstrate strength, to attract females, or the pleasurable aspects of making noise.

One objective of this study was to check if Barney or the other chimpanzees of the colony were capable of producing a performance similar to the one reported in Dufour et al. (2015) on a regular basis. Some individuals produced long and/or elaborated So-U bouts. Most So-U were contextualized, short and “unremarkable,” except maybe for some sexual displays reminiscent of human and bird courtship displays as illustrated in Supplementary Video 3. In this video, the male successfully attracted a female's attention by repeatedly hitting a large barrel with a rather slow and clearly audible tempo (with multiple repositioning of the barrel toward the female). Note that in the wild, sexual displays are more likely to involve branch breaking or leaf clipping (Nishida, 1980) rather than demonstrative drumming *per se* (Crockford and Boesch, 2005). In this respect, this video illustrates, potentially, an innovative use of drumming compared to wild chimpanzees. When focusing on the longest and most decontextualized So-U, we found interesting rhythmical patterns. However, most of these manipulations were constrained by the general configuration of the sound production: like, for example, hitting a barrel with a semi-circle trajectory against the wall (see Supplementary Video 1, for an example). The rhythmical element was not therefore entirely controlled by the chimpanzee, as it was in Barney's case. If Barney's solitary drumming bout had not been recorded by chance, this unique evidence of potential rhythmicity in chimpanzees would never have been brought to light. He did not repeat this feat during the study and may never do so again, making this recording all the more valuable.

At this point, we may question the adequacy of Arom's “decontextualization” criterion when evaluating musicality in animals (Arom, 2000). Indeed, human music is often contextualized (associated to rituals and social functions). The inclusion of this criteria sets the bar very high for sound production in animals to be considered music, and excludes many vocal sophistications heard in some bird songs. It also excludes some of the rhythmical sexual displays we recorded here. A more flexible use of Arom's criteria might therefore be needed to widen our understanding of animal musicality. Nevertheless, the structure of Barney's initial performance remains undeniably and intuitively recognizable as drumming, and conforms with the “higher order” criteria proposed by Arom (2000).

The many studies that explore the origins of music (e.g., including research presented in this special issue), are hampered by the limited amount of documentation describing instrumental sound production in apes. Although music appears to be within the grasp of chimpanzees, they have not yet taken the step to music *per se*. This modest contribution was designed to provide additional information about the use of objects and various media for sound production by chimpanzees, thus providing a starting point for further work along these lines (see also Ravignani et al., 2013). Further studies should attempt to investigate the type of attraction that instrumental noise making has on chimpanzees, including the refinement and leisureliness expressed while doing so. This should contribute to a better understanding of how music evolved.

## Author contributions

ES and VD designed the study, analyzed the data and wrote the manuscript. PG and VD collected the data. CP supervised and designed the data analysis.

## Funding

CP was funded by an ANR programme blanc (ANR 12 BSV7 0013 02).

### Conflict of interest statement

The authors declare that the research was conducted in the absence of any commercial or financial relationships that could be construed as a potential conflict of interest.
