# Supplementary material for: The Extraordinary Nature of Barney's Drumming: A Complementary Study of Ordinary Noise Making in Chimpanzees
Source: Front Neurosci. 2017 Jan 19;11:2. doi: 10.3389/fnins.2017.00002 (PMC5244740; doi:10.3389/fnins.2017.00002)
Supplement: Supplementary file 5 [file Presentation1.PDF]

Supplementary information

1. Supplementary methods

1.1 The chimpanzee colony

The studied colony was composed of 6 different captive social groups of common Chimpanzees (Pan Troglodytes) mostly captive-born apart from the founders. Most of them were *Pan troglodytes verus* subspecies.

1.2 Procedure for the analysis of rhythmicity

The observers (PG in phase one, & PG and VD in phase 2) stayed in the courtyard to collect data. When So-U could be filmed they were later analyzed for rhythmicity. A first step consisted in considering only the longest So-U (i.e superior to 20 sec). Within these So-U, we checked whether the production of sound was continuous and clearly detectable for at least 20 consecutive inter-beat intervals using the software reaper. Within a So-U we delimited sequences as follow: first, after a pause of two seconds, we considered the following beats to be the beginning of the next sequence. Second, if sound pollution occurred (caused by wind or when the noise of the So-U was covered by background noise), the inaudible part of the sequence was not analyzed, and we considered the following audible part as a new sequence. To analyze rhythmical patterns, we only considered sequences with more than 20. The beating onset was detected using *Reaper software*. We used the transient detection settings tool (threshold detection was set at -24 dB and sensitivity at 25%, 60% and 75%) to find the onset of each beat and thus measure the inter-beat durations in each sequence.

The series of consecutive inter-beat duration were then analyzed per sequence using a portmanteau test, the Ljung-Box test (Supplementary Figure S2). This test can provide several p-values, with one for each lag. When working with time series, lag denotes the range between the equally spaced instants of measurement. Here our instants of measurement are the beats and the measure is the inter-beat duration. Hence when considering a given lag, for example the third lag, we consider the relationship between one inter-beat duration and the one measured 3 beats later. If the analysis leads to significant p-values, this indicates that the data are not drawn at random and that there is a pattern in the time series.

For the sequences where non-random patterns were detected with the Ljung-Box test, we ran an autocorrelation procedure in order to test the linear dependency between each consecutive inter-beat durations (Figure S3). Dependency up to lag 3, for example, indicates that whatever the position in the sequence, the duration of the next three lags can be predicted.

2. Supplementary results

2.1 Context analysis

2.1.1 Supplementary figure 1

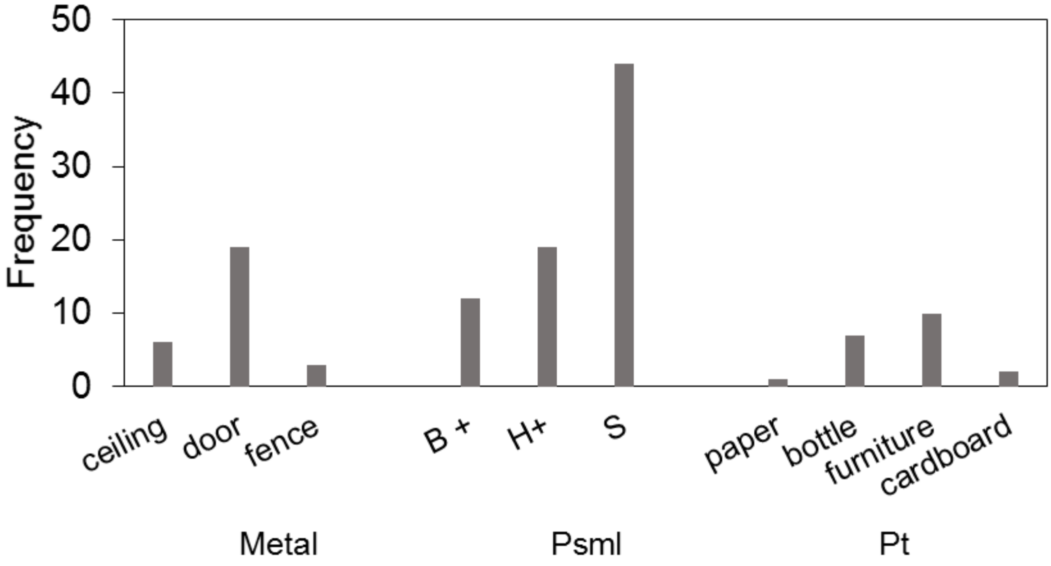

Supplementary Figure 1: Frequency of use of the various types of medium (with B+ for large barrel with or without a smaller size container, H+ for Half size barrel with or without a smaller size container, S for small container; Metal for metallic medium, Psml for small, medium and large plastic containers, Pt for all the other non-resonant and/or smaller objects).

2.2 Supplementary results of rhythmical analysis

Results of the Ljung-Box test are presented in Supplementary Figure S2. Patterns can be detected within up to 14 lags in the seven first sequences. Dennis seq 3.2 is significant until the lag 11, and the significance is only valid until lag 2 in Dennis seq 3.3.

Results of the autocorrelation test are presented in Supplementary Figure S3. Note that for Oscar seq 2.1, the alternation of long (above the central X-axis) and short lags (below the central X-axis) is highly noticeable. The technic used here is the half-circling technique that means that it took longer for the barrel to complete the trajectory in one direction compared to the other. Paul seq 1 also shows a nice and long interdependency with alternating series of short and long durations.

Figure S2: Ljung-Box test results. The test returns statistically significant results when the vertical line remains lower than 0.05. Oscar seq 1 is globally significant over 14 lags.

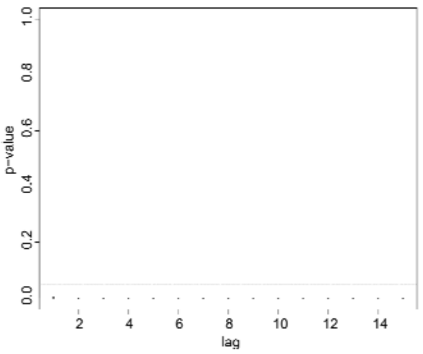

Freek seq 1

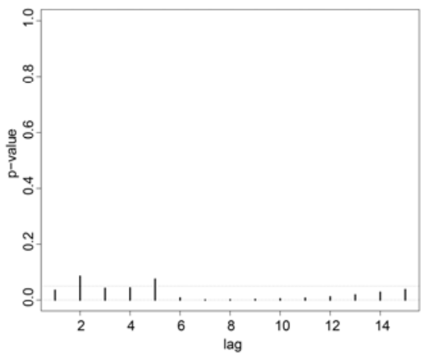

Oscar seq 1

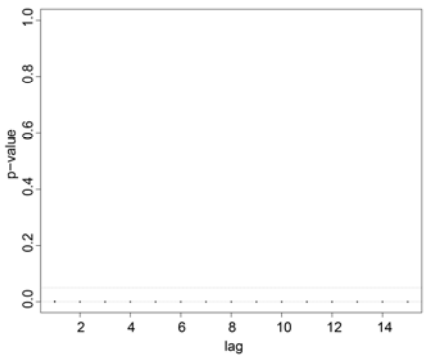

Oscar seq 2.1

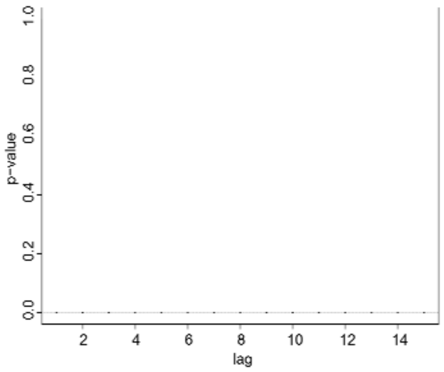

Paul seq 1.2

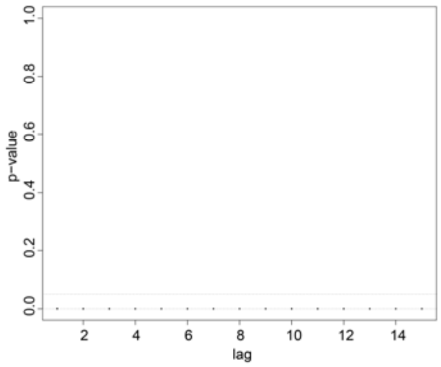

Dennis seq 2.2

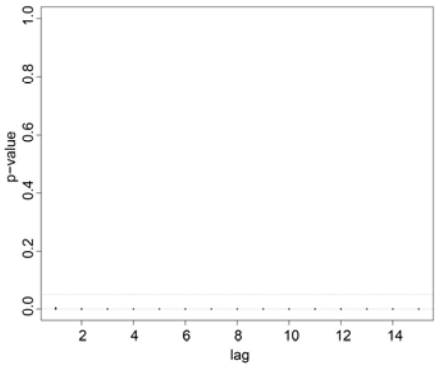

Ruben seq 1.1

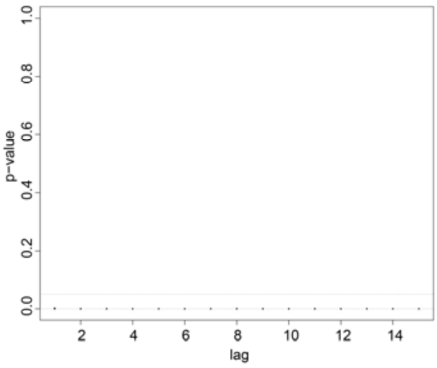

Dennis seq 3.1

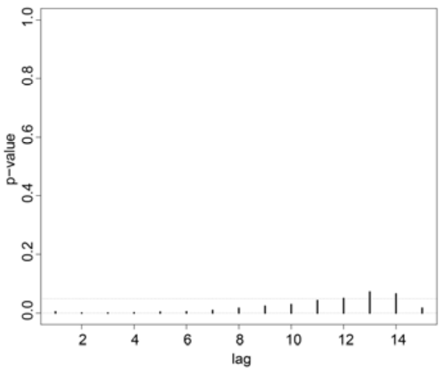

Dennis seq 3.2

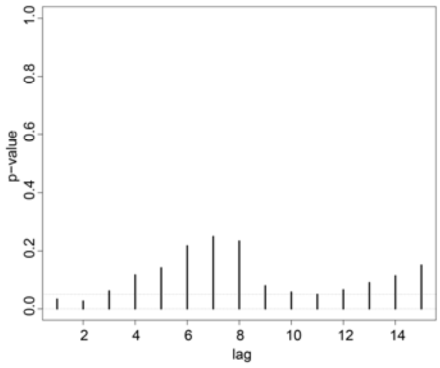

Dennis seq 3.3

Figure S3: Autocorrelation test results. Autocorrelation tests results for the 9 sequences with non-random rhythmical patterns showing the level of significance (Y-axis) according to the lag considered (X-Axis). The test returns statistically significant results when the vertical line reaches or crosses the blue lines. Whatever the position in the sequence, the duration of the next inter-beat duration is significantly correlated for up to 2 lags in Freeq seq 1; 1 lag in Oscar seq 1, more than 24 lag in Oscar seq 2.1; etc.

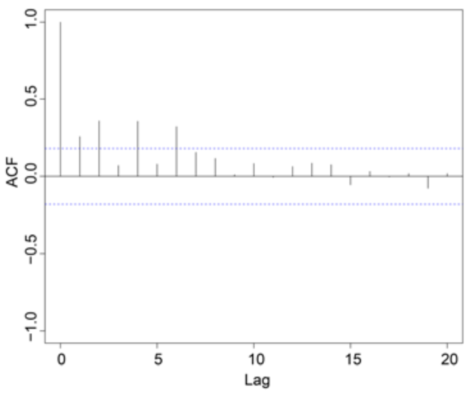

Freek seq 1

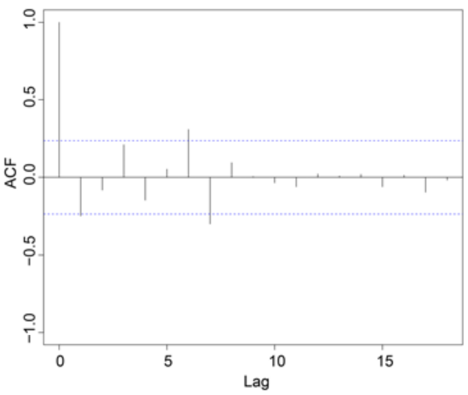

Oscar seq 1

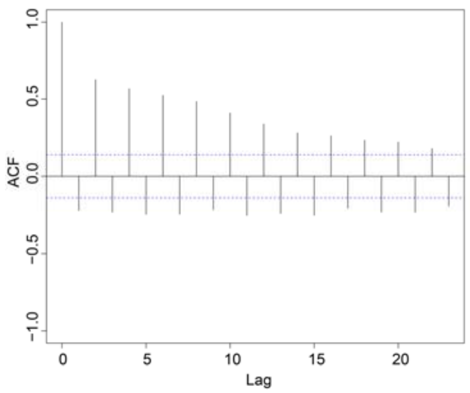

Oscar seq 2.1

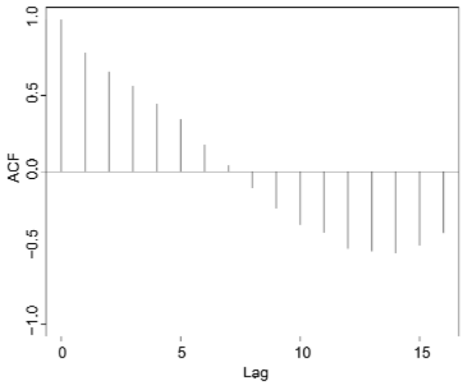

Paul seq 1.2

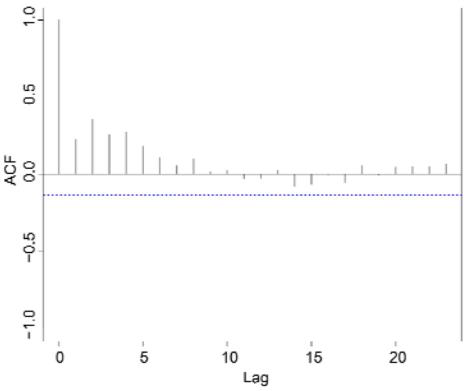

Dennis seq 2.2

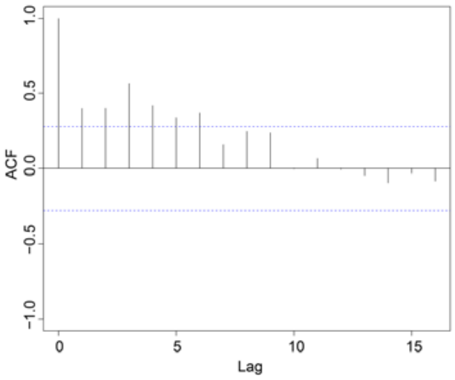

Ruben seq 1.1

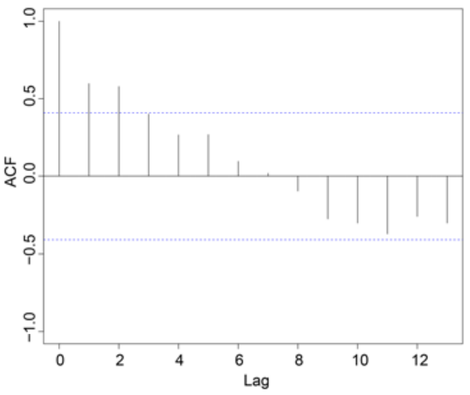

Dennis seq 3.1

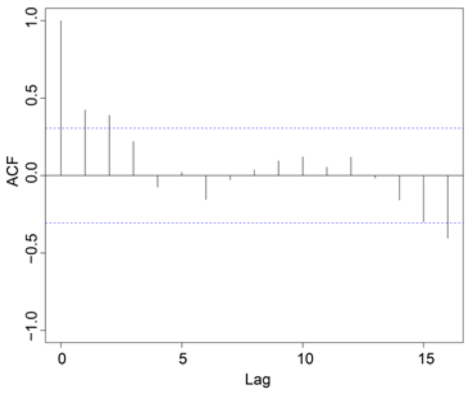

Dennis seq 3.2

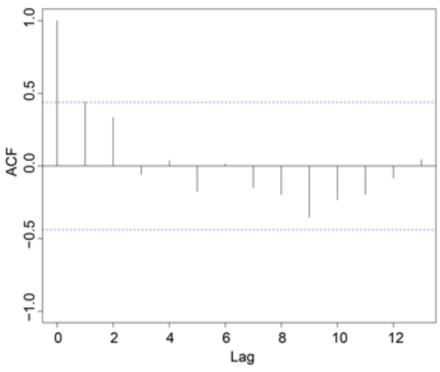

Dennis seq 3.3
